# GFR Evaluation Among Patients with Cancer: Insights and Clinical Implications

**DOI:** 10.3390/cancers18030351

**Published:** 2026-01-23

**Authors:** Alok Arora, Parnika Shukla, Vinay Srinivasan, Leyre Zubiri Oteiza, Zachary LeMense, Ginseng Vang, Paul E. Hanna

**Affiliations:** 1 Division of Nephrology, Department of Medicine, Medical College of Wisconsin, Milwaukee, WI 53226, USA; 2 Division of Nephrology, Department of Medicine, Cooper University Hospital, Cooper Medical School of Rowan University, Camden, NJ 08103, USA; 3 Dana Farber Cancer Institute, Boston, MA 02215, USA

**Keywords:** GFR estimation, patients with cancer, CKD-EPI 2021, Cystatin C, measured GFR

## Abstract

Accurately assessing the glomerular filtration rate (GFR) is critical in patients with cancer for acute kidney injury diagnosis, chemotherapy selection, drug dosing, and clinical trial eligibility. Yet, traditional equations such as Cockcroft–Gault and MDRD fail due to multiple physiological changes specific to this vulnerable population. Cancer-related sarcopenia, creatinine secretion blockade, and total body volume fluctuations may lead to inaccurate GFR estimations. This ultimately leads to undertreatment of underlying malignancy, overdosing of nephrotoxic therapies with adverse effects, and excluding patients from clinical trials unnecessarily. The 2024 KDIGO guidelines as well as the American Society of Onconephrology position statement recommend the use of combined GFR equation such as CKD-EPI 2021 that utilizes both cystatin C and creatinine to improve GFR estimation accuracy. Direct GFR measurement via exogenous filtration markers should be pursued in high-risk patients when precise values are warranted. This review highlights current challenges associated with GFR evaluation in patients with cancer and outlines clinical implications as well as recent recommendations for optimal clinical practice.

## Simple Summary

Diagnosis of acute kidney dysfunction, adjusting lifesaving therapy dosing, or enrolling in cancer clinical trials are key elements in onconephrology, the overlap between cancer and kidney care. These elements primarily rely on an accurate assessment of kidney function in patients with cancer. Patients with cancer were excluded from deriving conventional kidney function estimating formulas, which often fail to provide a reliable estimate in malignancy. This is due to changes in muscle mass, body fluids, and how creatinine is excreted in cancer. These changes may lead to inaccurate kidney function assessments, impacting important treatment decisions and risk-averse events. Optimized approaches based on the most recent guidelines that utilize alternative kidney biomarkers, or direct measurement of kidney function, are warranted to improve care.

## 1. Introduction 

Patients with cancer represent a special heterogeneous population in whom accurate estimates of glomerular filtration rate (GFR) are critical. Many cancer therapeutics have a narrow therapeutic range that requires close monitoring, while others are primarily renally eliminated. Thus, accurate dosing of cancer therapeutics is necessary to avoid toxicity and ensure patients are not excluded from clinical trials needlessly. This narrative review discusses some of the challenges of GFR estimation in this population and outlines clinical implications as well as recent recommendations to optimize clinical practice in onconephrology.

Serum creatinine is widely used in clinical settings primarily due to its ease. It is released continuously by skeletal muscles, filtered freely by the glomerulus, but also 20–30% is secreted by the proximal tubule, depending on a variety of factors [1]. Older creatinine-based equations, including the 1976 Cockcroft and Gault (CG) equation and the 1998 Modification of Diet in Renal Disease (MDRD) equations, are not validated in patients with cancer but are still commonly used in clinical practice [2,3]. The CG equation, for instance, uses expert opinion alone without clinical evidence to assign a sex factor of 0.85 to women to account for lower muscle mass [4]. Secondly, the CG equation was derived using 24 h urine collections as a reference for measured creatinine clearance (CrCl) and thus estimates CrCl and not eGFR (eGFR). The MDRD equation was developed in 1999 to estimate GFR and not CrCl, but it still suffers from the vast array of non-GFR determinants that can influence creatinine.

### 1.1. Biological Factors

Numerous factors can influence creatinine, including muscle mass, dietary protein intake, age, and sex. GFR estimation is further complicated by the understanding that GFR is not fixed but, in fact, declines with normal aging. As shown in Table 1, certain estimates demonstrate lower expected GFR values with older age, with modest sex-related differences. Fixed eGFR thresholds may therefore classify older adults with physiological age-related decline as having CKD, underscoring the importance of interpreting eGFR in conjunction with clinical context and the knowledge that GFR is variable.

Patients with cancer often develop sarcopenia; the reported prevalence varies from 11 to 44% in patients with liver, breast, gastric, and colorectal cancer, and up to 79% of patients with small cell lung cancer [5,6]. Higher dietary protein intake from animal sources has also been associated with higher serum creatinine, thereby resulting in lower eGFR when creatinine-based equations are used [7]. Furthermore, patients with cancer may appear obese yet have evidence of CT-defined sarcopenia due to an imbalance between adipose and skeletal muscle tissue. In a prior study, Hanna et al. showed that CT-defined sarcopenia and increased adiposity were both independently associated with large eGFR discordance, further contributing to biologic factors [8].

### 1.2. Clinical Factors

Medications can also impact serum creatinine due to their secretion by the proximal tubule. Numerous classes of anti-cancer medications, such as tyrosine kinase inhibitors (TKIs) and their subclasses, cyclin-dependent kinase (CDK 4/6) inhibitors, and poly(ADP-Ribose) polymerase (PARP) inhibitors, inhibit renal tubule transporters of creatinine secretion [9,10,11]. Other medications, including cimetidine, trimethoprim, and dolutegravir, also interfere with creatinine secretion by the proximal tubule and can result in a serum creatinine elevation [12]. Dysproteinemias have also been shown to interfere with the serum creatinine assay, when measured by both the enzymatic and Jaffe methods, resulting in a falsely elevated value [13]. In these cases, GFR should be estimated using other markers or measured to avoid inaccuracies based on serum creatinine measurement alone.

## 2. Cystatin C Overview

**Figure 1:**
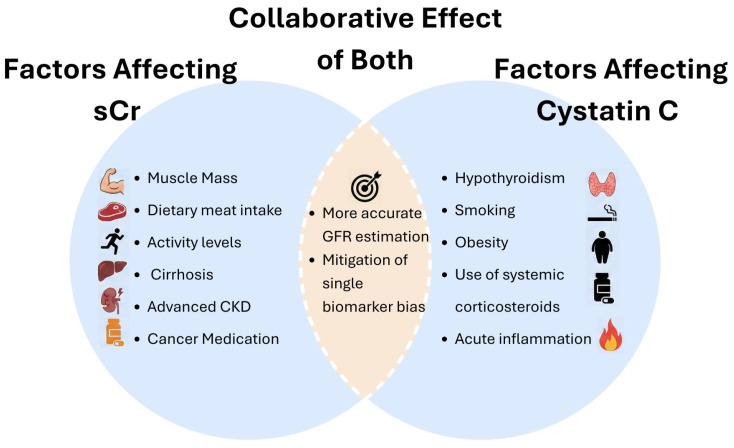
Venn diagram of factors that can collaboratively influence serum creatinine and cystatin C affecting eGFR.

Cystatin C is an endogenous inhibitor of lysosomal cysteine proteinases. It is filtered by the glomeruli and then reabsorbed and catabolized by the proximal tubule’s epithelial cells, with a small percentage excreted in the urine [14]. It is produced by all nucleated cells and not readily affected by changes in muscle mass, diet, sex, or age. It is, however, elevated in inflammatory settings, thyroid disease, obesity, and with corticosteroid usage [15]. In malignancy, however, cystatin C is expressed in cells of various solid organ tumors, and patients with cancer may have varying degrees of thyroid dysfunction or corticosteroid exposure, resulting in changing measurements [16]. Factors affecting both creatinine and cystatin C are illustrated in Figure 1.

## 3. Newer Equations to Estimate GFR

The 2009 CKD-EPI equation was based on serum creatinine and was believed to be more accurate than the MDRD equation [17]. In 2012, the CKD-EPI workgroup created two new equations, the CKD-EPI Cystatin C and CKD-EPI Creatinine-Cystatin C. Although they did not recommend replacing serum creatinine in routine practice, the workgroup indicated that using Cystatin C would be helpful in confirming decreased GFR as estimated by serum creatinine [18]. In 2021, race-free CKD-EPI equations that combined creatinine and Cystatin C were developed; these equations were not only more accurate but also led to smaller differences between black and non-black participants [19]. Common equations used to estimate GFR, along with assessment of bias and accuracy, are shown in Table 2.

Although these equations have improved the accuracy of eGFR, patients with cancer were typically not included in these cohorts. Most recently, the European Kidney Function Consortium (EKFC) developed a full age spectrum equation to estimate GFR; however, no black patients were included in this cohort [21]. Another EKFC equation, replacing creatinine with Cystatin C, included black patients and had a scaling factor for Cystatin C; the authors concluded that this equation improved the accuracy of eGFR compared to that of other commonly validated equations [22].

## 4. Measurement of GFR Using Exogenous Filtration Markers

Measurement of GFR using exogenous filtration markers is the gold standard to determine kidney function [23]. Many exogenous markers have been used, although the most common are iohexol and chromium-51 ethylenediaminetetraacetic acid (51Cr-EDTA); specifically, plasma clearance of iohexol has become the most commonly used method [24]. Although these tests offer the greatest accuracy, they are not universally available, and cost barriers also make them impractical for routine use. The 2024 Kidney Disease Improving Global Outcomes (KDIGO) Clinical Practice Guidelines for the Evaluation and Management of Chronic Kidney Disease recommend measuring GFR when eGFRcr-cys may be inaccurate due to non-GFR determinants in conditions such as cancers with high cell turnover or when eGFRcr or eGFRcys are widely divergent. They also recommend measuring GFR when greater accuracy is needed to dose medications that have a narrow therapeutic index or risk of serious toxicity [25].

## 5. Performance of eGFR Equations in Patients with Cancer

Given the limited number of cancer patients in the cohorts used to derive the above equations, recent studies have attempted to identify the best equations to estimate GFR in this population. Historically, CG has been commonly used for drug dosing by the oncology community, as many drug dosing guidelines were developed prior to the development of the CKD-EPI equation [26]. A systematic review in 2023 included adults and children with acute and chronic illnesses, including cancer. Five out of the 26 identified studies included patients with cancer. In this population, eGFRcr-cys improved GFR estimation, although the overall performance remained poor in many studies, leading the authors to recommend measuring GFR more frequently [27].

Another study from Brazil compared the accuracy of the CKD-EPI equations to the CG equation in 1200 adults with solid organ cancers who had GFR measured using 51Cr-EDTA. The authors found that CG was the least accurate equation to estimate GFR, while CKD-EPIcr-cys was the most accurate [20]. In another study of over 2600 patients with cancer, Fu et al. compared the performance of GFR equations using plasma iohexol clearance as a reference method; the authors of this study also concluded that the CKD-EPIcr-cys combined equation was the most accurate in this patient population [28].

## 6. Kidney Injury Biomarkers

Various biomarkers of kidney injury, especially acute tubular injury, have been identified but are yet to be widely adopted in clinical practice. Neutrophil gelatinase-associated lipocalin (NGAL) was initially detected in neutrophil granules and is upregulated several-fold in the first few hours after ischemic injury in mouse models and can be detected as early as 3 h in the kidney and urine after cisplatin administration [29]. Kidney injury molecule-1 (KIM-1) is a transmembrane glycoprotein and a marker of ischemic injury in the proximal tubules. Studies have shown that both biomarkers can show a rise several weeks prior to the elevation in serum creatinine, predicting sub-clinical nephrotoxicity with a high degree of sensitivity and specificity [30]. Although these biomarkers may predict subclinical nephrotoxicity prior to the rise in serum creatinine, they are not universally available, limiting their clinical utility. Additionally, they have not been validated in large studies in patients with cancer. Therefore, in our opinion, noted elevations in these biomarkers should be followed by measuring GFR using exogenous filtration markers to guide therapeutic decision-making more precisely.

## 7. Pediatric Patients

Estimating GFR in the pediatric oncologic population is particularly challenging due to patients generally having lower muscle mass, inflammation, and the difficulty of applying formulas for eGFR initially developed for healthy adults. In the pediatric population, equations using creatinine alone to estimate GFR tend to overestimate GFR and do not perform as well compared to equations containing cystatin C, although studies vary widely in the eGFR equations used and the measured GFR standards. Furthermore, small sample sizes and the inclusion of patients with primarily solid organ malignancies limit generalizability [31]. In this specific patient population, the best-performing equations to estimate GFR were those that combined cystatin C and creatinine, resulting in accuracy rates of greater than 80% [32,33]. Similar to the adult population, mGFR should be used when needing to establish a high degree of precision for therapeutic decision-making [31], with current recommendations to use either iohexol or ^99m^Tc-DTPA plasma clearance for measurement.

## 8. Chemotherapy Dosing and Eligibility

**Figure 2:**
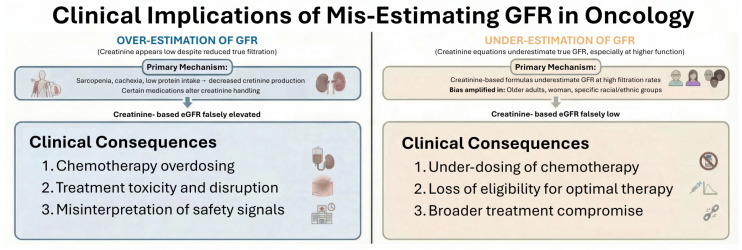
Clinical implications of mis-estimating GFR in oncology.

Accurate assessment of kidney function is central to oncologic care because renal clearance determines the disposition, toxicity, and therapeutic window of many anticancer agents, yet in patients with cancer, glomerular filtration rate is exceptionally difficult to estimate. Profound alterations in body composition, systemic inflammation, protein-energy wasting, hepatic dysfunction, tumor-driven cytokine changes, and exposure to medications that modify creatinine production or tubular secretion all undermine the reliability of serum creatinine as a filtration marker. As a result, creatinine-only equations frequently deviate from true GFR, creating a wide margin for error in chemotherapy dosing and eligibility determinations. These inaccuracies are not simply numerical discrepancies; they directly shape treatment decisions and clinical outcomes [26]. Figure 2 summarizes the clinical consequences of misestimating kidney function in cancer patients.

Among the most consequential clinical issues is the overestimation of kidney function. This arises predominantly in patients with sarcopenia, cachexia, and reduced dietary protein intake, in whom serum creatinine remains deceptively low despite significantly reduced glomerular filtration. When creatinine-based equations such as Cockcroft–Gault, MDRD, or even CKD-EPIcr overestimate GFR, chemotherapy doses are calculated based on a renal capacity the patient does not actually possess. The consequences are well characterized with cisplatin, a drug whose dose-limiting nephrotoxicity is closely linked to cumulative exposure. Individuals with cancer-related muscle wasting may appear eligible for cisplatin based on creatinine levels, yet exhibit marked reductions in true renal clearance, predisposing them to acute kidney injury, profound electrolyte losses, and long-term declines in kidney function [34].

Similar problems arise with high-dose methotrexate, where delayed clearance due to impaired GFR can lead to severe mucositis, hepatotoxicity, myelosuppression, and life-threatening systemic accumulation [35]. Several targeted therapies, including tyrosine kinase inhibitors, PARP inhibitors, antibody-drug conjugates, and drug conjugates, also demonstrate steep exposure-toxicity gradients, such that modest overestimation of GFR can translate into clinically meaningful toxicity.

Conversely, underestimation of kidney function is equally problematic and often leads to patients being denied optimal or potentially curative therapy. Creatinine-based formulas tend to underestimate GFR at higher levels of true kidney function, resulting in patients being incorrectly labeled as “renally impaired.” This has major implications in settings where specific GFR thresholds dictate treatment selection.

For example, cisplatin-based regimens demonstrate superior efficacy over carboplatin in several malignancies, yet many patients are steered toward carboplatin because their eGFR is underestimated, compromising their likelihood of cure or durable response [36]. Underestimation also affects dosing of carboplatin itself; since the Calvert formula incorporates GFR directly, small errors in eGFR lead to substantial reductions in delivered AUC and consequently in therapeutic intensity [34]. Beyond cytotoxic chemotherapy, inaccurate GFR estimates may exclude patients from stem cell transplantation, CAR-T cell therapy, or clinical trials with strict renal criteria. These effects are especially pronounced in older adults, the largest demographic in oncology, where age-associated muscle loss amplifies the bias of creatinine-based assessments [37].

The clinical implications of these inaccuracies extend beyond single-agent dosing. Mis-eGFR disrupts treatment sequencing, delays time-sensitive therapies, alters dose intensity, and can lead to premature discontinuation based on perceived but inaccurate renal “contraindications.” Patients incorrectly deemed ineligible for therapies such as cisplatin or transplant conditioning regimens may receive less effective alternatives, altering the trajectory of disease control.

**Figure 3:**
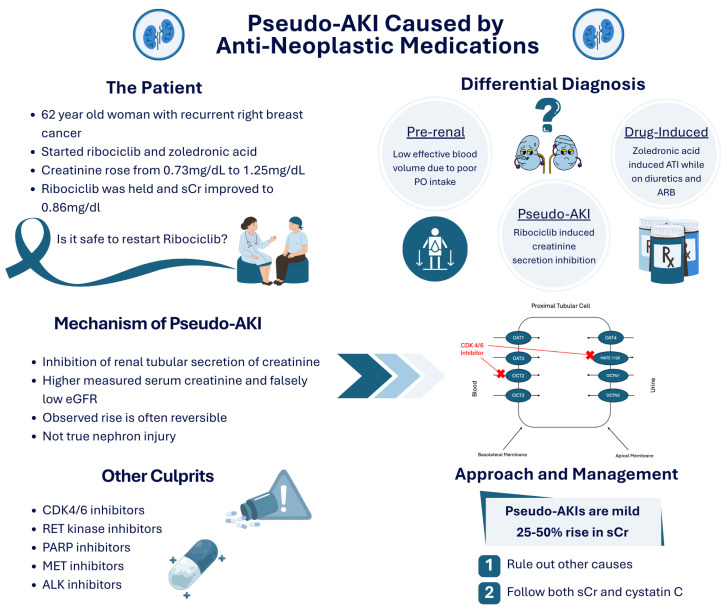
Representative case of a cancer patient with a pseudo-acute kidney injury due to inhibition of renal tubular secretion of creatinine from ribociclib, a CDK 4/6 inhibitor, leading to underestimation of GFR. CDK 4/6 inhibitors and PARP inhibitors are defined as inhibitors of cyclin-dependent kinase and poly(ADP-ribose) polymerase, respectively.

Conversely, patients whose GFRs are falsely elevated may experience avoidable toxicity, hospitalization, or irreversible renal injury. A case representation of a patient receiving ribociclib for breast cancer with subsequent development of a pseudo-acute kidney injury is described in Figure 3. In a disease domain where timing, intensity, and regimen selection are essential to achieving optimal outcomes, even modest misestimation can shift the balance between cure and failure [26].

These challenges underscore the growing importance of cystatin C in onconephrology. Unlike creatinine, cystatin C is minimally influenced by muscle mass, nutritional status, or most cancer-related metabolic alterations. Equations incorporating cystatin C, particularly the CKD-EPIcr-cys equation, consistently demonstrate improved accuracy and reduced bias relative to creatinine-only equations across diverse populations, including patients with cancer [20]. In oncologic practice, cystatin C effectively reduces misclassification around clinically important thresholds, restoring eligibility for therapies previously withheld due to falsely low creatinine-based estimates. At the same time, cystatin C frequently unmasks occult renal impairment in sarcopenic individuals whose creatinine appears normal, thereby preventing inadvertent overdosing. This bidirectional correction, mitigating both over- and underestimation, makes cystatin C particularly valuable in a population prone to extreme variations in muscle mass and metabolic state [38].

In situations where creatinine and cystatin C estimates diverge significantly, or where treatment decisions hinge on narrow renal thresholds, direct measurement of GFR using exogenous filtration markers provides the most definitive assessment. Although measured GFR is not universally available, its selective use is justified in scenarios involving highly nephrotoxic agents, strict eligibility criteria, or curative-intent regimens. Measured GFR offers clarity when eGFR values straddle cisplatin thresholds, when high-dose methotrexate is planned, or when renal function determines eligibility for transplant or investigational therapy [24]. Additionally, mGFR should be used instead of eGFR when: kidney function is in a non-steady state, such as acute kidney injury (AKI), during pregnancy, if there is a large discrepancy between eGFRcr and eGFRcys, and when receiving kidney replacement therapy, as endogenous markers may provide unreliable estimates in these situations. In such cases, mGFR prevents unwarranted exclusion, avoids dangerous overdosing, and ensures that therapeutic decisions reflect true physiologic kidney function rather than the limitations of imperfect estimation equations.

Collectively, these considerations highlight that precise GFR assessment is not an ancillary detail but a central determinant of safe, effective, and equitable cancer care. Misestimation produces avoidable toxicity, insufficient dose intensity, treatment delays, and denial of potentially life-saving therapies. As oncology continues to refine treatment paradigms and integrate renal thresholds into increasingly protocolized pathways, the nephrology community has a critical role in promoting accurate filtration assessment. Wider adoption of cystatin C, preferential use of modern validated GFR equations, and strategic use of measured GFR when appropriate are practical, evidence-based strategies that meaningfully improve outcomes for patients with cancer [39].

## 9. Drug Development and Regulatory Implications

Renal function assessment plays a pivotal role in oncology drug development, yet the methodologies used to evaluate kidney function in clinical trials have not evolved at the same pace as our understanding of GFR estimation. Most investigational and approved oncology agents undergo early-phase pharmacokinetic studies that stratify patients by renal function, and nearly all pivotal clinical trials incorporate renal thresholds as inclusion or exclusion criteria. These thresholds, however, were established at a time when creatinine-based estimates, most commonly Cockcroft–Gault or MDRD equations, were used almost universally, despite their known limitations in populations with altered muscle mass, systemic inflammation, or fluctuating physiologic states [26]. As a result, a central tension has emerged: although accurate GFR estimation is integral to patient safety, dosing, and regulatory guidance, the tools used to define renal function in oncology trials often lack the precision required to support these critical decisions.

Inadequate renal assessment has wide-reaching implications for trial eligibility. Since GFR cutoffs frequently serve as gatekeepers to study enrollment, variability in the method used to estimate GFR can result in inconsistent and sometimes unjustified exclusions. Two patients with identical true kidney function may be classified differently depending on whether GFR is calculated using the Cockcroft–Gault, MDRD, CKD-EPI equation, or a combined CKD-EPI creatinine–cystatin C equation [40]. This inconsistency disproportionately affects older adults, women, individuals with cancer-associated sarcopenia, and certain racial and ethnic groups, all of whom are more likely to have their renal function underestimated by creatinine-only equations. The result is a systematic under-representation of these populations in clinical trials, limiting the generalizability of oncology drug development and narrowing the evidence base for real-world practice.

Misestimation of renal function also complicates regulatory decision-making. Dose adjustments listed in drug labels are often derived from pharmacokinetic studies that categorize renal impairment using imprecise creatinine-based equations. When these methods overestimate kidney function, early-phase studies may underestimate toxicity risk, leading to insufficient dose-reduction strategies in the approved label. Conversely, when renal function is underestimated, as frequently occurs with MDRD or Cockcroft–Gault, regulatory agencies may endorse overly conservative dosing recommendations that restrict the use of therapies without clear evidence of harm [41]. These discrepancies are especially consequential for agents with narrow therapeutic windows, such as certain tyrosine kinase inhibitors, antifolates, and antibody–drug conjugates, for which even modest inaccuracies in renal function categorization can translate into clinically significant differences in drug exposure.

An additional challenge arises from therapies that alter creatinine handling without affecting true GFR. Several TKIs and immunotherapies inhibit tubular creatinine secretion or cause inflammation-driven increases in serum creatinine [42]. In trials that rely solely on creatinine-based equations, these pharmacodynamic effects may be misinterpreted as nephrotoxicity, prompting unnecessary treatment interruptions, dose reductions, or discontinuation. This misclassification obscures true safety signals and complicates the interpretation of renal adverse events. Incorporating cystatin C or measured GFR in early-phase studies would help distinguish true reductions in filtration from benign creatinine shifts, thereby improving both patient management and regulatory clarity [43].

A persistent gap in the regulatory landscape is the scarcity of high-quality data for patients with chronic kidney disease (CKD). Because many clinical trials exclude individuals with moderate CKD, the safety and efficacy of numerous oncology therapies in this large patient population remain inadequately characterized [44]. Clinicians are therefore left to extrapolate dosing recommendations from limited or indirect evidence, often erring on the side of caution. Improving the precision of renal function assessment-whether through cystatin C, combined equations, or measured GFR-could meaningfully expand trial participation among patients with CKD and strengthen the evidence base guiding their treatment [45].

## 10. Conclusions 

**Figure 4:**
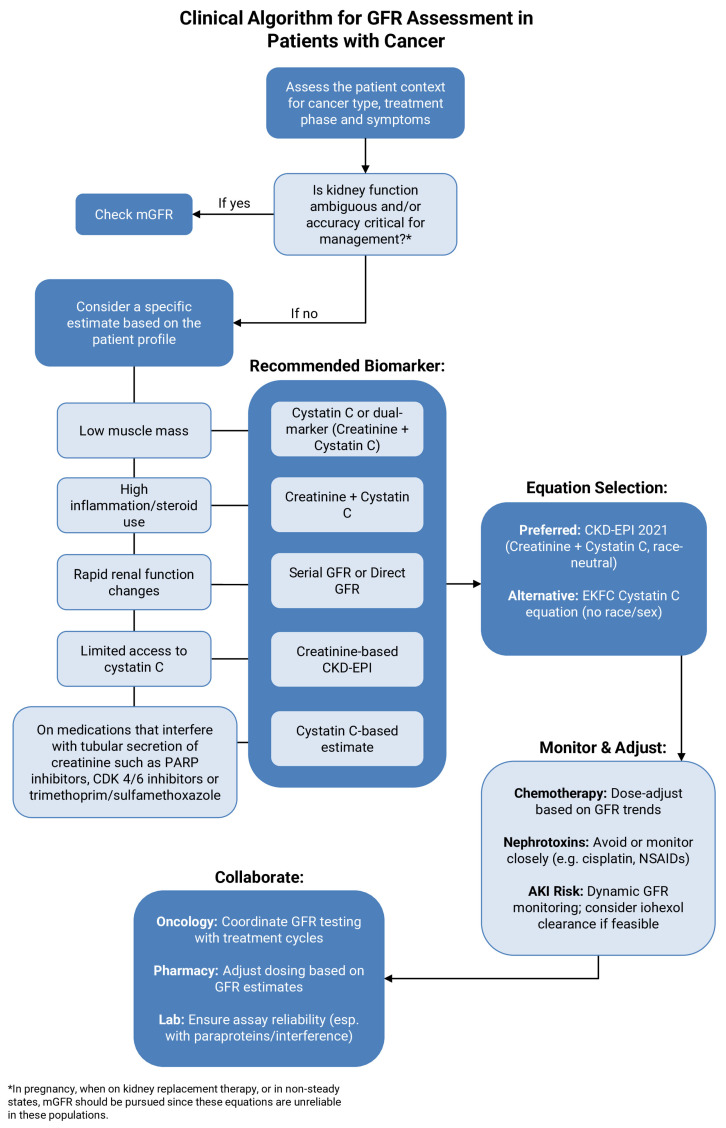
Clinical algorithm for GFR assessment in patients with cancer.

Together, these considerations highlight the urgent need for standardized renal assessment practices in oncology drug development [46]. The American Society of Onco-Nephrology’s (ASON) position statement provides a foundation for standardization and advocates for using the validated CKD-EPIcr-cys equation for decision-making regarding anti-cancer therapy and dose adjustment. It further recommends avoiding using non-validated equations, such as CG, and measuring GFR via exogenous filtration markers when eGFR results in borderline eligibility for therapies or clinical trials [43,47]. Similarly, the International Consensus Guideline on Anticancer Drug Dosing in Kidney Dysfunction (ADDIKD) recommends the eGFRCKD-EPI equation as the most accurate way of estimating GFR, while directly measuring GFR in certain clinical situations where the CKD-EPI equation may be unsuitable, and measured GFR is also preferred for initial dosing of certain medications such as cisplatin, carboplatin, and methotrexate [48]. Figure 4 delineates a clinical algorithm for GFR assessment in patients with cancer across various patient profiles.

Transparent reporting of the method used to estimate GFR, routine incorporation of cystatin C in early-phase pharmacokinetic analyses, wider use of CKD-EPI creatinine–cystatin C for eligibility and dose modeling, and strategic use of measured GFR for drugs with high nephrotoxicity risk would collectively improve trial design, regulatory decision-making, and real-world treatment equity. As oncology increasingly embraces precision medicine, the tools used to measure renal function must also become more precise, ensuring that treatment eligibility and safety assessments reflect true physiological capability rather than the limitations of outdated estimation formulas.

## Figures and Tables

**Table 1:** Age- and sex-adjusted estimates of glomerular filtration rate (eGFR) in healthy adults and corresponding KDIGO GFR categories. * Normal ranges reflect population-based estimates in healthy individuals without albuminuria or structural kidney disease.

Age Group (Years)	Expected Mean eGFR in Men (mL/min/1.73 m^2^)	Expected Mean eGFR in Women (mL/min/1.73 m^2^)	Approximate Normal Range *	KDIGO GFR Category	Clinical Interpretation
20–29	110–120	105–115	≥90	G1	Normal or high renal function
30–39	105–115	100–110	≥90	G1	Normal renal function
40–49	95–105	90–100	85–100	G1-G2	Normal aging-related decline
50–59	85–95	80–90	75–95	G2	Mildly decreased, often age-appropriate
60–69	75–85	70–80	65–90	G2	Expected decline with aging
70–79	65–75	60–70	55–85	G2-G3a	May overlap with CKD by fixed thresholds
≥80	55–65	50–60	45–80	G3a	Often physiological if stable and without kidney damage

**Table 2:** Bias and accuracy assessment of common equations used in the estimation of GFR.

GFR Estimation	Marker	Effect on eGFR	Bias (mGFR − eGFR) mL/min/1.73 m^2^	Accuracy
Equation				(1 − P30)
Cockcroft-Gault (3)	sCr	Overestimation eGFR	−8.1 (−9.4 to −6.7)	24.9 (22.4 to 27.3)
MDRD (4)	sCr	Overestimation eGFR	−4.8 (−6.0 to −3.6)	18.2 (16.0 to 20.3)
CKD-EPI (5)	sCr	Overestimation eGFR	−8.1 (−8.9 to −7.1)	19.1 (16.8 to 21.2)
CamGFRv2 (6)	sCr	Underestimation eGFR	6.1 (5.3 to 6.9)	7.2 (5.7 to 8.7)
CKD-EPI (7)	Cys-C	Underestimation eGFR	4.6 (3.7 to 5.5)	12.3 (10.3 to 14.3)
CKD-EPI (7)	sCr + Cys-C	Overestimation eGFR	−2.0 (−2.6 to −1.1)	7.8 (6.3 to 9.4)

Common equations for estimation of GFR, their associated biomarkers, and assessment of bias and accuracy compared to mGFR chromium-51-labeled ethylenediamine tetraacetic acid (51Cr-EDTA) clearance in 1200 patients with solid tumors. sCr, creatinine; Cys-C, cystatin-C; MDRD, modification of diet in renal disease; CKD-EPI, chronic kidney disease epidemiology collaboration; CamGFRv2, Cambridge University Hospitals National Health Service (NHS) Foundation Trust; P30, proportion of estimates within 30% of mGFR. Data and 95% confidence intervals are shown. Adapted from Costa E Silva et al. [20]. Bias is defined as the median difference between estimated and measured GFRs. Accuracy was assessed with P_30_ (proportion of patients whose estimated GFRs are within 30% of measured GFRs).

## Data Availability

No new data were created or analyzed in this study. Data sharing is not applicable to this article.

## Abbreviations

The following abbreviations are used in this manuscript:

ADDIKD	Anticancer Drug Dosing in Kidney Dysfunction
ASON	American Society of Onco-Nephrology
CDK	Cyclin-Dependent Kinase
CG	Cockcroft and Gault
CKD	Chronic Kidney Disease
CKD-EPI	Chronic Kidney Disease Epidemiology Collaboration Equation
CrCl	Creatinine Clearance
Cr-EDTA	Chromium-51 ethylenediaminetetraacetic acid
CT	Computed Tomography
eGFR	Estimated Glomerular Filtration Rate
EKFC	European Kidney Function Consortium
GFR	Glomerular Filtration Rate
KDIGO	Kidney Disease Improving Global Outcomes
MDRD	Modification of Diet in Renal Disease
PARP	Poly (ADP-Ribose) Polymerase
TKI	Tyrosine Kinase Inhibitor
